# Association between triglyceride-glucose index and the risk of stroke among moyamoya patients: a single-center retrospective study

**DOI:** 10.3389/fneur.2025.1547627

**Published:** 2025-03-14

**Authors:** Chi Zhang, Kai Guo, Hong Xu, Gang Kong, Chuanghong Liu

**Affiliations:** First People’s Hospital of Changshu City, Changshu, Jiangsu, China

**Keywords:** triglyceride-glucose index, Moyamoya disease, stroke, insulin resistance, atherosclerosis of the arteries

## Abstract

**Background:**

Moyamoya disease increases the risk of strokes by impairing cerebral circulation, significantly affecting patients’ quality of life. Despite the profound implications of this condition, there is a limited body of research exploring the factors that contribute to strokes in patients with Moyamoya disease. This study aims to evaluate the predictive value of the triglyceride-glucose index (TyG index) in forecasting stroke events among individuals diagnosed with moyamoya disease.

**Methods:**

A total of 84 patients diagnosed with Moyamoya disease were treated at Changshu First People’s Hospital from 01 January 2019 to 31 October 2024 were included in this study. We systematically collected and analyzed their clinical histories, laboratory test results, and baseline data. The patients were classified into two groups based on their history of cerebral stroke. Subsequently, we conducted a comparison and analysis of the TyG index between these two groups.

**Results:**

The TyG index observed in patients with moyamoya disease who have a history of cerebral stroke was significantly elevated compared to that of patients without such a history. This difference was found to be statistically significant (*p* < 0.05). Furthermore, regression cubic splines analysis indicated a noteworthy linear relationship between the TyG index and the occurrence of cerebral stroke events.

**Conclusion:**

In individuals diagnosed with Moyamoya disease, the TyG index has been shown to have a significant correlation with the risk of cerebral stroke. Furthermore, it has the potential to serve as an effective predictor for the occurrence of stroke.

## Introduction

Moyamoya disease (MMD), also known as spontaneous basilar ring occlusive disease or abnormal basal vascular network disorder, is a rare and chronic cerebrovascular condition. It is characterized by the progressive stenosis of the major intracranial arteries, resulting in the formation of a network of small, abnormal blood vessels. Notably, these vessels exhibit a distinct smoke-like appearance during angiographic imaging (1). The etiology of Moyamoya disease is not yet fully understood; however, it is associated with significant neurological complications stemming from compromised cerebral circulation. These complications can manifest as both ischemic and hemorrhagic strokes (2). The occurrence and progression of strokes have a significant impact on the daily lives of patients with Moyamoya disease. It is essential to investigate the factors that contribute to stroke in these patients and to implement targeted interventions aimed at reducing stroke incidence. Such efforts can improve clinical outcomes and overall prognosis for individuals affected by this condition. However, it is important to note that research in this area is currently limited (3, 4).

Insulin resistance (IR) is recognized as an independent risk factor for mortality and significant disability following a stroke (5). Historically, the hyperinsulinemic-euglycemic clamp (HEC) method has been considered the gold standard for diagnosing IR. However, the clinical implementation of this procedure has become increasingly challenging due to its significant cost, complex operational requirements, and the extensive time commitment involved (6). In recent years, the triglyceride-glucose index (TyG index) has emerged as a promising compound metabolic index. Research indicates that it is a straightforward and reliable alternative for evaluating insulin resistance (IR). Furthermore, the TyG index has demonstrated significant associations with the risk of cardiovascular and cerebrovascular events, along with various arterial diseases (7, 8).

This study aims to evaluate the potential of the TyG index as a predictive tool for stroke in patients diagnosed with Moyamoya disease. We collected clinical and laboratory data from 84 patients with Moyamoya disease, including 35 asymptomatic individuals and 49 patients with a history of stroke. The TyG index was calculated using fasting triglyceride and glucose levels, and its association with stroke risk was analyzed using logistic regression models, ROC curve analysis, and restricted cubic splines. Our results revealed that the TyG index was significantly higher in patients with a history of stroke compared to asymptomatic patients, demonstrating a strong linear association with stroke risk and suggesting its potential as a predictive biomarker in Moyamoya disease.

## Methods

### Study population

This retrospective study collected medical history and clinical examination data from patients with Moyamoya disease admitted to the Department of Neurosurgery at Changshu First People’s Hospital between01 January 2019 and 31 October 2024. A total of 84 patients with Moyamoya disease participated in this study, which included 35 asymptomatic patients and 49 patients who had experienced previous strokes. The inclusion criteria were based on the diagnostic standards outlined in the “Moyamoya disease: diagnosis and interventions” (9). Exclusion criteria: (1) Cerebrovascular changes resembling smog caused by hyperthyroidism, autoimmune diseases, brain radiotherapy, head trauma, and other conditions; (2) Patients who have previously undergone cerebrovascular reconstructive surgery.

In this study, the definition of stroke (10) encompasses both hemorrhagic and ischemic types. Hemorrhagic stroke was defined as a sudden neurological deficit caused by the rupture of a cerebral blood vessel, resulting in bleeding into the brain parenchyma (intracerebral hemorrhage, ICH) or the subarachnoid space (subarachnoid hemorrhage, SAH). Diagnosis was confirmed using non-contrast computed tomography (CT) or magnetic resonance imaging (MRI), which revealed hyperdense areas indicative of hemorrhage. Ischemic stroke was defined as a sudden neurological deficit caused by the interruption of blood flow to a part of the brain, resulting in tissue ischemia and infarction. Ischemic stroke subtypes were classified as thrombotic, embolic, or lacunar based on the underlying mechanism of arterial occlusion. Diagnosis was confirmed using non-contrast computed tomography (CT) or magnetic resonance imaging (MRI), with diffusion-weighted imaging (DWI) used to identify early ischemic changes.

Asymptomatic patients (11) were defined as those without a history of stroke, transient ischemic attack (TIA), or any other neurological symptoms attributable to moyamoya disease at the time of diagnosis. These patients were identified during routine health check-ups or evaluations for unrelated conditions.

### Data collection and definition

Clinical medical records were systematically collected from patients, encompassing key demographic information such as age and gender, as well as relevant medical histories, including diabetes and hypertension. In addition, early morning fasting venous blood samples were obtained, with laboratory analyses conducted within 12 h of patient admission. TyG index = ln [fasting triglyceride (mg/dl) × fasting glucose (mg/dl)/2] (12).

### Statistical analysis

Quantitative variables were reported as mean ± standard deviation. Differences between groups were assessed using either ANOVA or the Kruskal-Wallis test based on data distribution. Categorical variables are reported in terms of counts and percentages, and their analysis is conducted using chi-square tests.

To assess the relationship between the TyG index and the risk of stroke, a logistic proportional hazards regression model was employed to calculate odds ratios (ORs) and 95% confidence intervals (95% CIs). Three statistical models were developed by varying the adjustment for covariates. Model 1 includes no adjustments. Model 2 incorporates adjustments for gender and age. Model 3 provides additional adjustments for hypertension, diabetes, serum creatinine (SCR), uric acid (UA), total cholesterol (TC), high-density lipoprotein cholesterol (HDL-C), and low-density lipoprotein cholesterol (LDL-C). The Receiver Operating Characteristic (ROC) curve was utilized to evaluate the predictive performance of the TyG index regarding stroke risk. Additionally, a fully adjusted restricted cubic spline (RCS) analysis was conducted to explore the dose–response relationship between the TyG index and stroke risk in patients diagnosed with Moyamoya disease.

Additionally, subgroup analyses were conducted to determine if the association between the TyG index and stroke risk varied across different demographics, including gender, age, hypertension, and diabetes. Interaction analyses were conducted to investigate potential variations in stroke risk across the identified subgroups. To ensure the robustness of our findings, a sensitivity analysis was performed, calculating an E-value based on Model 3. This analysis aimed to determine the minimum strength of association between unmeasured confounders and the TyG index that could potentially explain the observed association with stroke risk. All statistical analyses were performed utilizing R version 4.2.2. For the purposes of this study, *p* values below 0.05 were deemed statistically significant.

## Results

### Baseline characteristics

The baseline characteristics of the participants are summarized in Table 1. In the subgroup with a history of stroke (*N* = 49), the following measurements were found to be significantly elevated compared to those in the subgroup without a history of stroke (*N* = 35): fasting triglycerides (1.62 ± 0.85 mmol/L, *p* = 0.008), fasting blood glucose (6.59 ± 2.48 mmol/L, *p* = 0.019), TyG index (8.88 ± 0.67, *p* < 0.001), and high-density lipoprotein (1.12 ± 0.28 mmol/L, *p* = 0.008). Both groups exhibited no statistically significant differences in the levels of creatinine, urea, total cholesterol, and low-density lipoprotein. The analysis revealed that there were no significant differences in the distribution of sex, hypertension, and diabetes among the groups.

**Table 1:** Patient demographics and baseline characteristics.

Characteristic	Stroke history			*p*-value
	Overall, *N* = 84	Non-stroke, *N* = 35	Stroke, *N* = 49	
Gender				0.415
Male	38 (45.2%)	14 (40.0%)	24 (49.0%)	
Female	46 (54.8%)	21 (60.0%)	25 (51.0%)	
Age	49.61 ± 11.246	49.34 ± 12.570	49.80 ± 10.330	0.862
Hypertension				0.795
Yes	47 (56.0%)	19 (54.3%)	28 (57.1%)	
No	37 (44.0%)	16 (45.7%)	21 (42.9%)	
Diabetes				0.511
Yes	74 (88.1%)	32 (91.4%)	42 (85.7%)	
No	10 (11.9%)	3 (8.6%)	7 (14.3%)	
FTG mmol/L	1.43 ± 0.80	1.17 ± 0.67	1.62 ± 0.85	0.008
FBG mmol/L	6.14 ± 2.21	5.52 ± 1.61	6.59 ± 2.48	0.019
SCR Umol/L	62.58 ± 15.35	63.40 ± 13.83	62.00 ± 16.46	0.674
UA mmol/L	5.10 ± 1.60	4.84 ± 1.17	5.28 ± 1.84	0.182
TC mmol/L	4.09 ± 1.20	4.18 ± 1.44	4.03 ± 1.01	0.601
HDL-C mmol/L	1.20 ± 0.34	1.32 ± 0.37	1.12 ± 0.28	0.008
LDL-C mmol/L	2.27 ± 0.78	2.12 ± 0.71	2.38 ± 0.82	0.137
TyG	8.68 ± 0.67	8.39 ± 0.57	8.88 ± 0.67	<0.001

FBG, fasting blood-glucose; FTG, fasting triglycerides; HDL-C, high-density lipoprotein cholesterol; LDL-C, low-density lipoprotein cholesterol; TC, total cholesterol; TyG, triglyceride-glucose; SCR, serum creatinine; UA, Urea.

### Relationship between stroke risk and TyG index

A total of 49 patients had a documented history of stroke. Logistic regression analysis demonstrated that each one-unit increase in the triglyceride glucose index (TyG), analyzed as a continuous variable, was significantly associated with an increased risk of stroke across all three models. In the unadjusted Model 1, the odds ratio (OR) was calculated at 3.95 (95% CI: 1.68, 9.28; *p* = 0.002), suggesting a significant association. This strong association persisted in Model 2, which adjusted for sex and age, yielding an OR of 4.04 (95% CI: 1.65, 9.91; *p* = 0.002). Furthermore, in Model 3, which accounted for additional covariates, the OR was recorded at 3.95 (95% CI: 1.25, 12.51; *p* = 0.020). These findings highlight the robustness of the association across multiple analytical models. In the analysis of the quartile test utilizing the TyG as a categorical variable with Q1 as the reference group, the odds ratio for Q2 in model 3 is 5.15 (95% confidence interval: 0.99, 26.63; *p* = 0.051). For Q3, the odds ratio is 11.20 (95% CI: 1.84, 68.04; *p* = 0.009), and for Q4, it stands at 15.00 (95% CI: 2.08, 108.38; *p* = 0.007). These findings indicate that, compared to the baseline of Q1, the risk of stroke is elevated by a factor of 5.15 for Q2, 11.2 for Q3, and 15 for Q4. The trend of *p*-values across all models was found to be statistically significant (*p* < 0.001). This suggests that the observed relationship continues to be significant even after making the necessary adjustments for covariates (Table 2).

**Table 2:** Association between the TyG index and stroke incidence in a population with Moyamoya.

Characteristic	Event, *n*	Model 1		Model 2		Model3	
		OR (95% CI)	*p*-value	OR (95% CI)	*p*-value	OR (95% CI)	*p*-value
TyG index (per 1 unit)	49	3.95 (1.68,9.28)	0.002	4.04 (1.65,9.91)	0.002	3.95 (1.25,12.51)	0.02
TyG index quartile							
Q1	5	Ref		Ref		Ref	
Q2	12	4.80 (1.25,18.42)	0.022	5.85 (1.41,24.39)	0.015	5.15 (0.99,26.63)	0.051
Q3	16	8.53 (2.16,33.73)	0.002	11.07 (2.39,51.19)	0.002	11.20 (1.84,68.04)	0.009
Q4	16	10.24 (2.47,42.37)	0.001	13.65 (2.85,65.42)	0.001	15.00 (2.08,108.38)	0.007
P for trend			<0.001		<0.001		0.008

OR, Odds Ratio; CI, Confidence Interval.

Model 1: no covariates were adjusted.

Model 2: adjusted for gender and ager.

Model 3: adjusted for gender, ager, hypertension, diabetes, SCR, UA, TC, HDL-C, and LDL-C.

### Subgroup and sensitivity analysis

**Figure 1:**
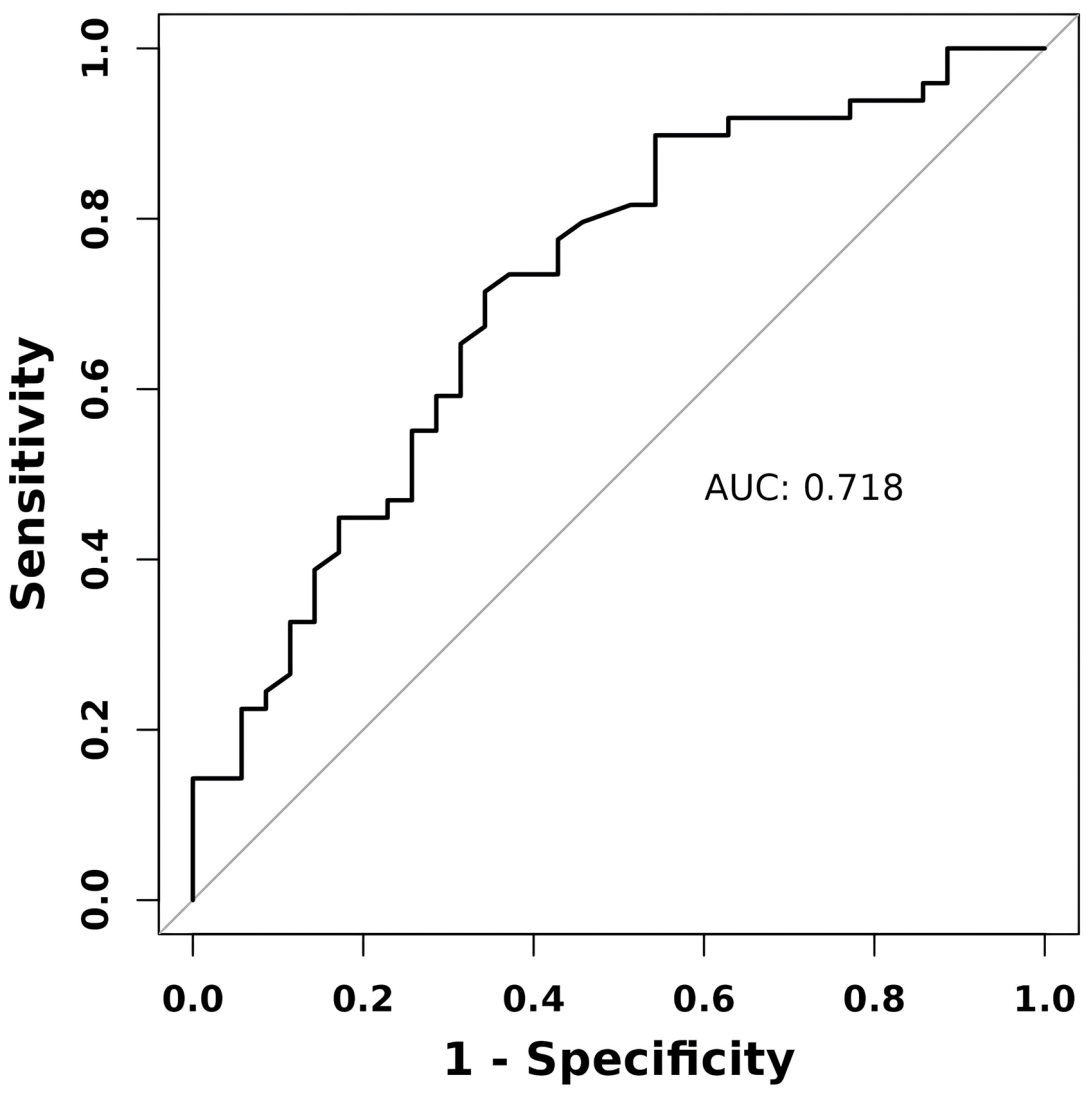
Receiver operating characteristic (ROC) curve analysis of the TyG index for predicting stroke in patients with Moyamoya disease.

**Figure 2:**
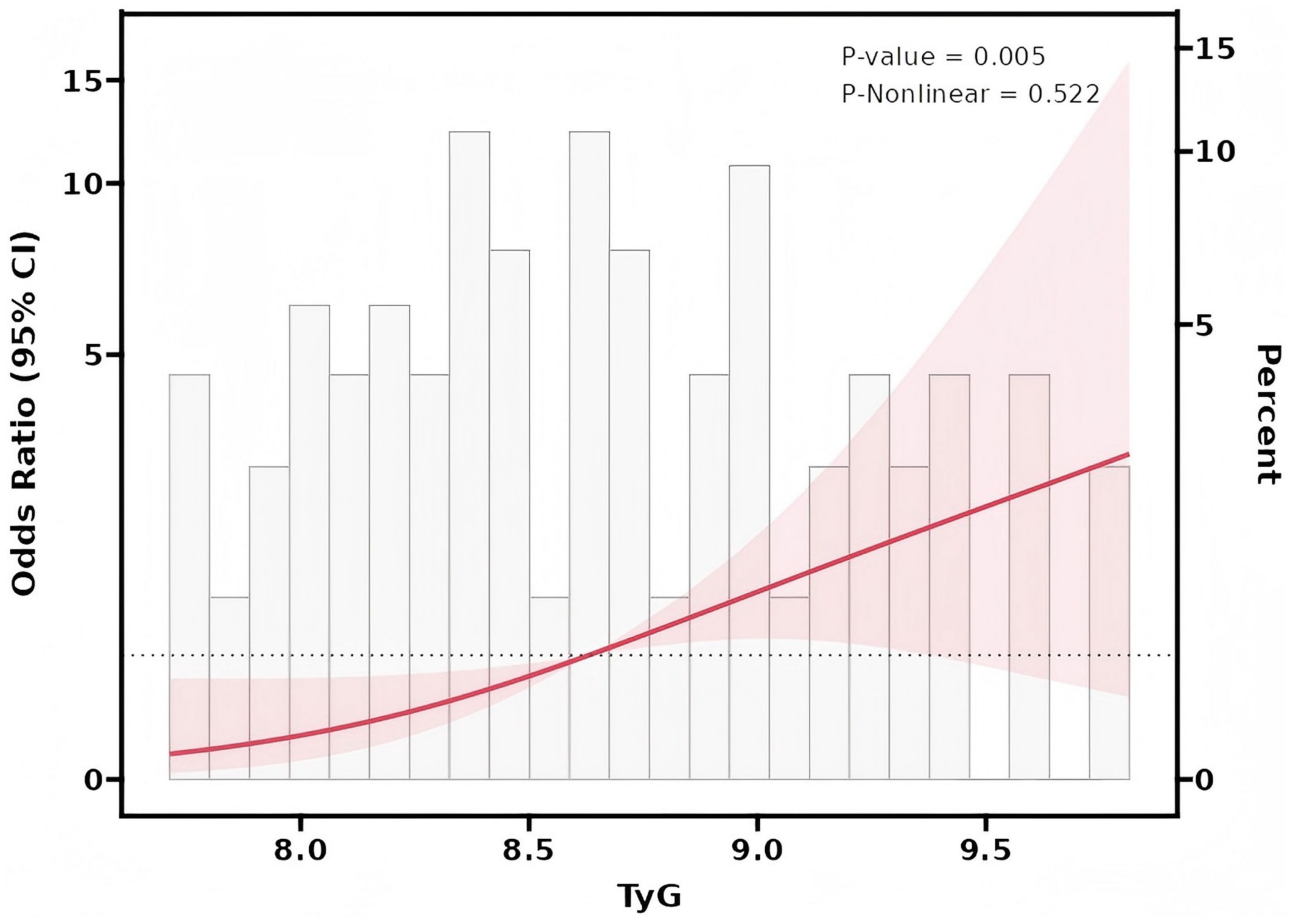
Radar cross section (RCS) statistical curve.

The value of the TyG index in predicting stroke among patients with moyamoya disease has been assessed using the ROC curve analysis. The results indicate an area under the curve (AUC) value of 0.718, signifying a strong predictive capability for this index in this patient population (Figure 1). Further analysis utilizing Restricted Cubic Splines (RCS) revealed a significant linear association between the TyG index and the occurrence of stroke events across all participants (*p* = 0.005, P for non-linear = 0.522) (Figure 2).

#### Sensitivity analysis

**Figure 3:**
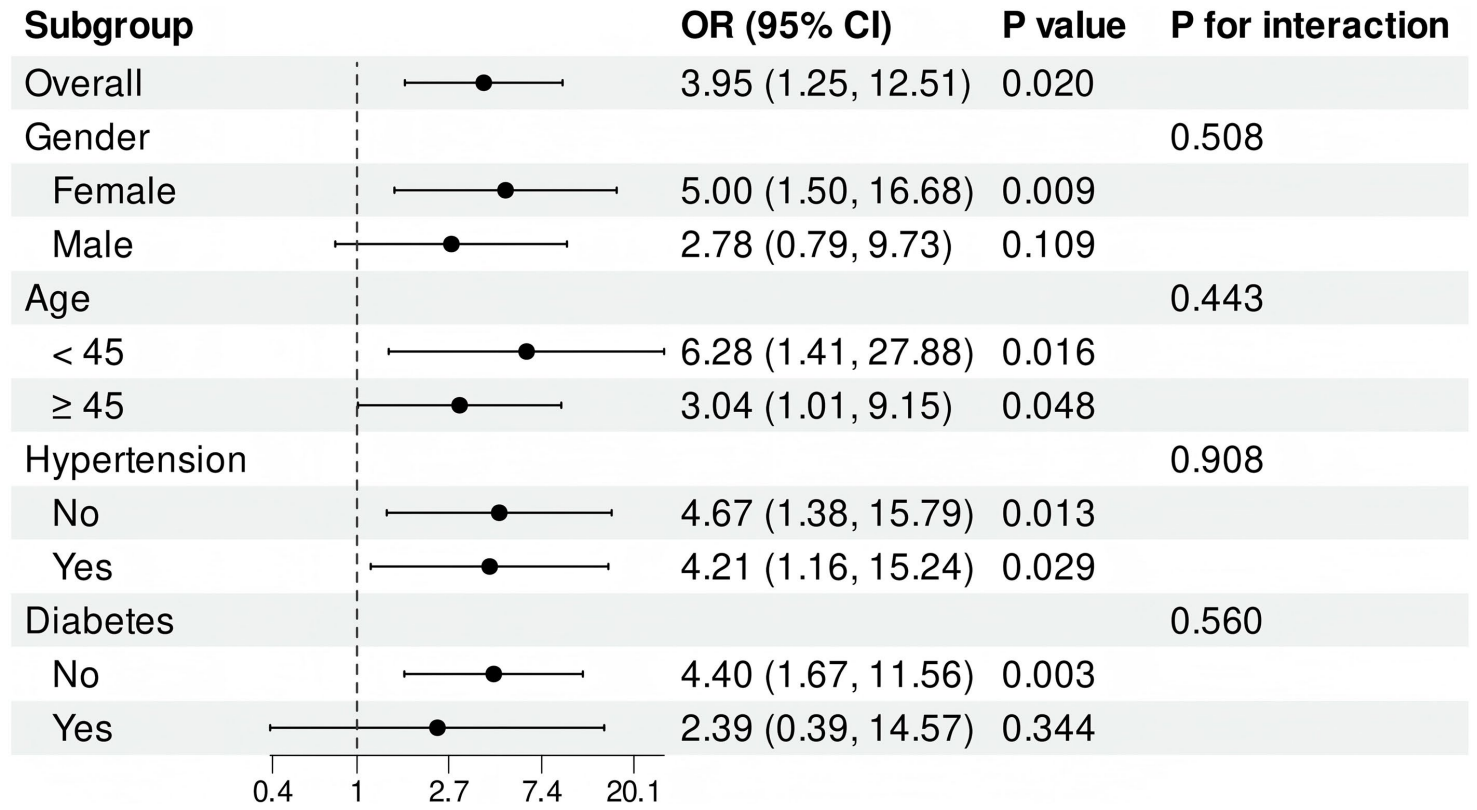
Subgroup analyses.

The calculation of an E value of 7.36 for TyG, based on model 3, indicates that only significant unmeasured confounders may account for the observed association. To enhance our understanding of the relationship between the TyG index and stroke incidence in patients diagnosed with Moyamoya disease, we conducted an analysis of the TyG index as a continuous variable. This analysis was performed within subgroups categorized by gender, age, hypertension, and diabetes. The findings demonstrate that elevated levels of the TyG index were consistently observed in female, non-diabetic subgroups, irrespective of age and the presence of co-hypertension. An interaction analysis was conducted to assess potential interactions between these subgroups and the TyG index. The results indicate that no significant interactions were found among the subgroups (Figure 3).

## Discussion

Moyamoya disease, first recognized by Japanese scholars in the 1850s, derives its name from the appearance of the abnormal network of blood vessels located at the base of the brain (13, 14). Presently, the etiology of Moyamoya disease is not fully understood. The condition is characterized by progressive stenosis and obstruction of the ends of the bilateral internal carotid arteries, as well as the anterior and middle cerebral arteries. Additionally, compensatory collateral vessels are formed that facilitate blood circulation (15). Moyamoya disease presents with a variety of initial forms, the most prevalent of which is stroke. This can manifest as both ischemic and hemorrhagic strokes (16, 17). Stroke represents one of the primary causes of significant disability in adults. Its impact extends beyond the affected individuals, profoundly influencing their long-term quality of life and imposing considerable challenges on their families and society as a whole (18). At this time, the understanding of the factors that affect stroke onset and prognosis in moyamoya disease is still quite limited. Further research is necessary to enhance our knowledge in this area.

Insulin resistance (IR) is a widely recognized concern within clinical practice and is a critical factor in the development and progression of cardiovascular and cerebrovascular diseases. In recent years, the triglyceride glucose index (TyG index) has emerged as a prominent research focus due to its status as a simple and reliable surrogate marker of IR (19). The TyG index serves as an effective indicator of the body’s metabolic state by integrating fasting triglyceride and blood glucose levels (20). It is closely associated with the risk of atherosclerosis as well as cardiovascular and cerebrovascular events (21).

Recent studies indicate that the TyG index plays a significant role in enhancing the instability of atherosclerotic plaques (22). This occurs through the activation of innate immune responses and the elevation of systemic inflammatory markers (23). The accumulation of advanced glycosylation end products (AGEs) is associated with the development of arteriosclerosis (24). A high TyG index has dual implications for cerebral health. On one hand, it is associated with a reduction in telangiectasia and the development of cerebral vasoconstriction, which can exacerbate cerebral ischemia through inflammatory processes (25). On the other hand, an elevated TyG index enhances the permeability of the blood–brain barrier, leading to brain edema, damage to vascular endothelial cells, and an increased risk of cerebral hemorrhage (26, 27). Furthermore, a high TyG index may be significantly associated with both the severity and number of intracranial artery stenosis, which may subsequently increase the risk of stroke (28). Extensive research has demonstrated a correlation between elevated TyG index levels and the progression of atherosclerosis (29, 30). This condition is a critical contributor to ischemic stroke, which subsequently impacts the course of stroke development (31). Limited research exists regarding the association between the TyG index and the incidence and progression of stroke in patients diagnosed with Moyamoya disease. This study represents the first investigation into the predictive value of the TyG index for stroke occurrence in patients with moyamoya disease. The findings reveal a significant correlation between the TyG index and the incidence of stroke in these patients, underscoring its considerable clinical relevance.

This study retrospectively examined a cohort of 84 patients diagnosed with Moyamoya disease, classifying them based on their history of stroke. The analysis indicated that the triglyceride-glucose (TyG) index was significantly elevated in patients with a prior stroke compared to those without. Additionally, when controlling for various covariates, including gender, age, total cholesterol, high-density lipoprotein (HDL), low-density lipoprotein (LDL), creatinine, and urea levels, the association between the TyG index and stroke remained statistically significant. The results of this study suggest that the TyG index may be an independent risk factor for stroke in patients diagnosed with Moyamoya disease. Additionally, the ROC curve analysis indicates that the TyG index is highly effective in predicting stroke, with an area under the curve (AUC) of 0.718. These findings further substantiate the clinical application potential of the TyG index.

The findings of this study present valuable new perspectives on the prevention and management of stroke in individuals diagnosed with Moyamoya disease. In patients exhibiting a high TyG index, early intervention and proactive treatment strategies may significantly mitigate the risk of stroke. Enhancing insulin sensitivity along with the regulation of lipid and glucose levels may effectively reduce the incidence of cerebrovascular events. Furthermore, early cerebral revascularization surgery has the potential to serve as an effective preventive measure for patients identified as high-risk.

It is important to note that this study has some inherent limitations, including individual variability in laboratory measurements, which may impact the outcomes. Additionally, the long-term predictive impact of continuous changes in the TyG index on the prognosis of Moyamoya patients was not evaluated. Future research should focus on larger sample sizes and extended follow-up periods to further substantiate these findings.

## Conclusion

In conclusion, this study presents, for the first time, a significant correlation between the TyG index and the occurrence of strokes in patients with moyamoya disease. This finding suggests that the TyG index may serve as a valuable predictor of stroke risk in this patient population. Consequently, it provides a novel perspective for the clinical management of moyamoya disease. Further research is warranted to confirm its clinical applicability and to investigate the underlying pathophysiological mechanisms.

## Data Availability

The original contributions presented in the study are included in the article/supplementary material, further inquiries can be directed to the corresponding author.
